# Cardiovascular Prevention: Current Gaps and Future Directions

**DOI:** 10.3390/diagnostics16010016

**Published:** 2025-12-20

**Authors:** Hélder Dores, José Ferreira Santos, Victor Gil, Pedro de Araújo Gonçalves

**Affiliations:** 1 Department of Cardiology, Hospital da Luz Lisboa, Luz Saúde, 1600-209 Lisbon, Portugal; victorgilmd@gmail.com (V.G.); paraujogoncalves@yahoo.co.uk (P.d.A.G.); 2 CHRC—Comprehensive Health Research Center, Associate Laboratory REAL (LA-REAL), 1099-085 Lisbon, Portugal; 3 NOVA Medical School, NOVA University Lisbon, 1069-061 Lisbon, Portugal; 4 CoLab Trials, 7000-811 Évora, Portugal; 5 Department of Cardiology, Hospital da Luz Setúbal, Luz Saúde, 2900-722 Setúbal, Portugal; 6 Católica Medical School, Sintra Campus, Estrada Octávio Pato, 2635-631 Lisbon, Portugal

**Keywords:** cardiovascular, prevention, risk stratification, digital, precision medicine

## Abstract

Cardiovascular Disease (CVD) remains the leading cause of morbidity and mortality worldwide. Despite significant advances in diagnosis and treatment, the global burden of CVD remains high, underscoring the crucial need for more effective and comprehensive prevention strategies. This narrative overview aims to critically evaluate the current pillars of cardiovascular prevention, identify the gaps in approaches and outline promising future directions. Challenges and barriers in lifestyle adherence and pharmacological management are addressed, while the increasing role of non-traditional and emerging risk factors is discussed. Future directions include maximizing the value of digital health to improve patient engagement and adherence, adopting precision medicine to refine risk stratification and implementing public health policies for population-level interventions. The optimization of cardiovascular prevention requires a multi-level approach that integrates clinical strategies with personalized solutions and environmental policies to ultimately reduce the global impact of CVD.

## 1. Introduction: The Global Burden of Cardiovascular Disease

Cardiovascular disease (CVD) represents the leading cause of death and disability worldwide, accounting for more than 18.6 million deaths annually, corresponding close to one-third of all global deaths, affecting individuals from different demographics and socioeconomic backgrounds [1,2,3]. Low- and middle-income countries are experiencing a disproportionate rise in CVD-related morbidity and mortality [2,4]. Most of the CVD burden is driven by atherosclerotic cardiovascular disease (ASCVD), with ischemic heart disease and stroke being the primary contributors. Additionally, other major conditions such as heart failure and atrial fibrillation are also concerning.

Beyond the clinical outcomes, CVD is a major source of direct and indirect healthcare costs [5,6]. The global trends of disability-adjusted life years (DALYs) and years of life lost due to CVD have continued to rise steadily, especially due to ischemic heart disease, stroke and hypertensive heart disease [7].

The fact that CVD remains a global health problem underscores that current strategies remain insufficient or are not being adequately implemented. Despite the considerable progress and expanding scope of preventive cardiology within modern CV medicine, major shortcomings persist in converting evidence-based knowledge into consistent, day-to-day clinical practice. Limitations within healthcare systems and global inequalities remain major challenges, while more tailored interventions at individual level should be implemented [8]. As the great majority of ASCVDs are preventable due to modification of traditional risk factors, accurate prevention is essential to overcome the consequences of these diseases [8,9]. Nevertheless, unhealthy lifestyle and modifiable CV risk factors remain highly prevalent and are often inadequately treated [7,10].

Core strategies for effective CV prevention should encompass early detection or diagnosis of CVD, precise risk stratification, individualized interventions, and continuous reassessment to optimize long-term outcomes [11]. The integration of emerging determinants and risk markers, such as genetics, circulating biomarkers, imaging and environmental characteristics, supported by digital technologies, incorporating artificial intelligence (AI), could improve CV risk stratification and lead to timely implementation of preventive and therapeutic measures [12,13]. Together, these strategies strengthen the adoption of precision medicine and enable the integration of novel pharmacological agents [14]. In this setting, the development of care pathways and multidisciplinary approaches encompassing the continuum of CV prevention are essential to address the challenges of individuals at risk or with already established CVD. Optimization of this approach has the potential to reshape how CVD is understood and managed in the near future.

Therefore, this narrative overview aims to examine the foundational components of CV prevention, delineate the major gaps that persist in contemporary practice, and discuss promising avenues that may guide research priorities and clinical implementation in the coming years.

## 2. Methodology and Literature Search

A narrative review was conducted, with relevant literature identified through a structured search of the PubMed database, focusing primarily on studies published within the last 10 years (1 January 2015 to 1 November 2025), with particular emphasis on evidence from the past 5 years. The search included original research articles, systematic reviews, narrative reviews, meta-analyses, and opinion papers deemed pertinent to the topic. In addition, the most recent European and American clinical guidelines related to cardiovascular (CV) prevention were directly reviewed and integrated into the analysis. After removing duplicates, titles and abstracts were screened for relevance, followed by full-text assessment of potentially eligible articles. When appropriate, additional references were identified through manual cross-referencing and backward citation searching.

The main topics of interest included current methodologies in CV prevention, commonly used risk scores, lifestyle and pharmacological preventive strategies, traditional and neglected CV risk factors, practical existing barriers and limitations in this field and potential future approaches, new directions, methodologies and tools to improving CV prevention. Being a narrative review, the selection and synthesis of the literature were conducted based on relevance to the research objectives and the authors’ critical appraisal of the available evidence. The search strategy combined terms related to CV prevention (e.g., “cardiovascular prevention”, “cardiovascular risk factors”, “traditional cardiovascular risk factors”, “neglected cardiovascular risk factors”, “cardiovascular risk stratification”, “cardiovascular risk modifiers”) with terms related to challenges and future directions (e.g., “gaps”, “barriers”, “limitations”, “new tools”, “challenges”, “future directions”, personalized interventions”, “digital tools”, “artificial intelligence”).

As a narrative review, this study has inherent limitations. Unlike systematic reviews, it lacks a systematic methodology for literature selection, potentially introducing selection bias and limiting reproducibility. Nevertheless, studies were rigorously selected based on relevance to the research objectives and critically appraised by the authors to provide a comprehensive overview of current gaps and future directions in CV prevention.

## 3. Core Pillars of Contemporary Cardiovascular Prevention

### 3.1. The Current Landscape: Critical Appraisal and the Need for Change

**Figure 1:**
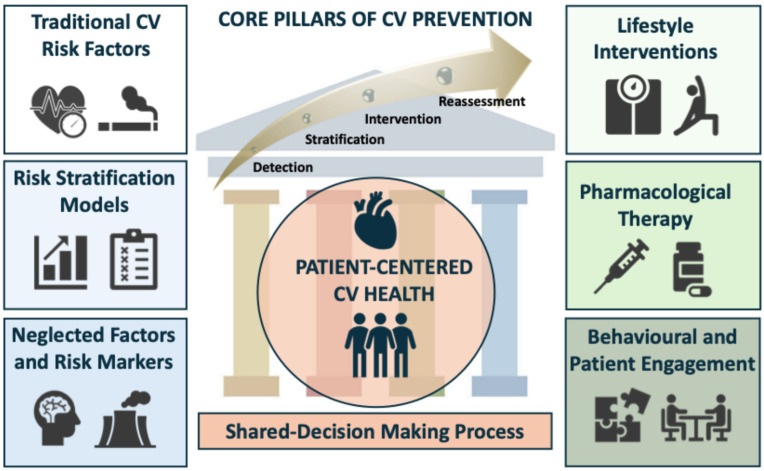
Core pillars of the current CV prevention. This diagram illustrates the prevention process as a continuous cycle (detection, stratification, intervention and reassessment) centered on patient CV health. Prevention is sustained by core intervention domains—lifestyle interventions, pharmacological therapy, behavioral and patient engagement, anchored by the assessment of traditional CV risk factors, risk stratification models, neglected factors and risk markers, within a shared decision-making framework.

Current CV prevention relies on an integrated approach that combines early detection and diagnosis of CVD, systematic risk classification, targeted interventions on modifiable risk factors with guideline-directed preventive therapies, and continuous reassessment to optimize long-term CV outcomes. Beyond established risk factors, prevention should also address non-traditional factors, risk markers and comorbidities associated with increased CV risk [11,15]. Individual risk stratification, supported by validated tools, enables the application of personalized strategies according to specific and evidence-based targets [11]. Figure 1 presents a global overview of the core pillars of current CV prevention. Despite the growing body of evidence in preventive cardiology, the practical implementation of guideline-based recommendations remains challenging due to barriers operating at multiple levels [16].

A healthy lifestyle throughout life remains the cornerstone of preventing CVD, particularly ASCVD. Lifestyle modification, including a balanced diet, regular physical activity, smoking cessation, weight management, and, when indicated, pharmacological treatment for hypertension, dyslipidemia, and diabetes, is essential to reduce CVD burden. Sustained adherence to physicians’ recommendations requires patient engagement and motivation, highlighting the importance of structured counseling and shared decision-making. Lifestyle modification requires implementation of measures at both individual and population levels to maximize their impact in clinical practice.

Refinements in CV risk stratification methodologies with inclusion of more objective parameters, definition of specific targets, and development of effective preventive and therapeutic strategies, are clearly promising. Current European Society of Cardiology (ESC) guidelines about CV prevention advocate a stepwise approach to treatment intensification to help physicians and patients achieving targets in alignment with individual profiles and preferences [11]. In practice, treatments should be initiated and progressively intensified within a shared decision-making process, supported by accurate and effective doctor-patient communication.

Strengthening preventive policies and improving real-world adherence remain central priorities in contemporary cardiovascular health, especially in the face of the projected rise in CVD burden predominantly driven by ASCVD [17]. Despite the persistence of unfavorable global statistics and continued disparities in access to specialized care, progress in preventive strategies offers grounds for optimism. Emerging evidence highlights the transformative potential of digital health technologies for continuous monitoring and timely intervention, alongside the growing impact of precision medicine, which leverages genomics and AI to tailor prevention to individual risk profiles. The transition from one-size-fits-all prevention strategies to an adaptable, personalized, and policy-driven model is crucial for reducing CVD incidence and mortality worldwide.

Although advances in CV prevention are promising, several known limitations, such as scarce long-term data for certain interventions, heterogeneous study designs, practical implementation barriers and the potential widening of inequities due to disparities in digital access, underscore the need for ongoing research and the pursuit of more effective, accessible and individualized preventive strategies.

### 3.2. Traditional Cardiovascular Risk Factors

The ongoing increase in CVD prevalence is largely driven by population aging and the persistence of lifestyle-related risk factors. Early identification of CV risk factors and timely diagnosis of CVD enable earlier intervention and are consistently associated with better clinical outcomes. Traditional risk factors, such as dyslipidemia, hypertension, smoking, and diabetes mellitus, remain central, as they are among the strongest contributors to ASCVD. Management of these risk factors combines lifestyle modification (healthy diet, regular physical activity, smoking cessation, and weight control) with pharmacological therapy when indicated (e.g., statins, antiplatelet agents, and antihypertensive drugs) [11].

The causal role of low-density lipoprotein cholesterol (LDL-C) and other apolipoprotein B (apoB)-containing lipoproteins in ASCVD is robustly demonstrated [18]. Non high-density lipoprotein cholesterol (non-HDL-C) shows a similar association with CV risk as LDL-C and provides information comparable to plasma apoB. Consequently, non-HDL-C has been incorporated into the Systematic Coronary Risk Evaluation 2 (SCORE2) and SCORE2-Older Persons (SCORE2-OP) algorithms endorsed by the ESC [19]. Despite strong evidence and clear guideline targets, most high- and very-high-risk patients fail to achieve recommended LDL-C levels, often due to underestimation of CV risk, therapeutic inertia, and underuse or under-intensification of lipid-lowering therapies [20].

High blood pressure is also a major risk factor for the development of multiple diseases, including ASCVD, heart failure, cerebrovascular disease, lower extremity arterial disease, chronic kidney disease and atrial fibrillation [21]. Global hypertension prevalence has remained high over the past decades, affecting a substantial proportion of adults worldwide. The number of people with hypertension doubled between 1990 and 2019 (from 650 million to 1.3 billion) and the great majority (three-quarters) live in low- and middle-income countries [22]. Although awareness, treatment, and control rates have improved in some regions, progress remains inconsistent, with many individuals remaining with uncontrolled blood pressure and approximately half of them being unaware of their condition. Sustained, large-scale public health strategies and effective implementation of guideline-directed therapy are urgently required to mitigate hypertension-related morbidity and mortality [23].

Cigarette smoking remains one of the leading preventable causes of death worldwide and is responsible for approximately half of all avoidable deaths in smokers, with a substantial proportion attributable to ASCVD. A lifelong smoker faces roughly a 50% probability of dying from a smoking-related cause and, on average, loses about 10 years of life expectancy, with CVD as a major contributor to this premature mortality [24]. Global tobacco use has declined over recent decades, driven by stronger tobacco-control policies, yet large regional, sex, and age disparities persist, and smoking prevalence remains high in many low- and middle-income countries. However, significant disparities remain by region, gender, and age, with men and certain regions, like the Eastern Mediterranean and Africa, maintaining high numbers of smokers [25]. While global smoking prevalence has fallen, smoking is still common and causes a significant health burden. The decline across the globe is shifting the epidemic progressively to low-middle income countries. Although the long-term effects on CV health are still not well established, all tobacco products that are alternatives to traditional cigarettes are associated with subclinical markers, particularly inflammatory markers potentially linked to CV harm [26]. Smoking is likely to remain a leading cause of preventable death throughout this century unless smoking cessation efforts can significantly and rapidly reduce the number of smokers, particularly in Asia [27].

Diabetes mellitus increases the risk of ASCVD by about two-fold, especially patients with type 2 diabetes mellitus that are more likely to have multiple CV risk factors, including dyslipidemia and hypertension [28,29]. Global diabetes prevalence in adults has doubled since 1990, rising from approximately 7% to 14% by 2022, affecting an estimated 828 million adults. The burden of diabetes and untreated diabetes is increasingly carried by low- and middle-income countries [30]. Suboptimal detection, treatment, and control of diabetes, together with limited access to comprehensive risk factor management, contribute substantially to excess CV risk in this population.

Taken together, these traditional CV risk factors (dyslipidemia, hypertension, smoking, and diabetes) remain highly prevalent and are closely linked to the development of CVD, mainly in individuals with poor control. When these risk factors cluster in the same individual, their effects are often synergistic, leading to a disproportionate multiplicative increase in ASCVD risk. Addressing this problem requires integrated public health strategies, improved risk factor management, and a broader shift towards preventive care to change the current scenario. This highlights the need for comprehensive, simultaneous management of multiple risk factors rather than isolated, single-factor interventions.

### 3.3. Cardiovascular Risk Stratification: SCORE2/SCORE2-OP

In general, the higher an individual’s absolute CV risk, the greater the benefit derived from comprehensive and intensive risk factor management, and the lower the number needed to treat to prevent a CV event over a given time horizon [31]. This principle underpins contemporary risk stratification with tools such as SCORE2 and SCORE2-OP, which aim to identify those individuals most likely to benefit from preventive interventions. Accurate and systematic CV risk assessment in the general population facilitates earlier identification of CV risk factors and is therefore essential for effective prevention. This risk assessment should be viewed as a continuous process with periodic reassessments.

The 2021 ESC prevention guidelines introduced significant advancements in risk assessment tools, particularly with the development of the SCORE2 and SCORE2-OP algorithms [11]. Further refinements in risk assessment tools were introduced in the 2023 ESC guidelines with the addition of SCORE2-Diabetes [32]. Additionally, the 2025 Focused Update of the 2019 ESC/EAS guidelines for the management of dyslipidaemias were also updated with these algorithms [15].

Risk scores should be applied in apparently healthy individuals, defined as those without established ASCVD or other comorbidities (e.g., chronic kidney disease and familial hypercholesterolemia), and estimate the 10-year risk of fatal and non-fatal CVD events (myocardial infarction and stroke). The SCORE2 should be applied for people aged 40–69 years and the SCORE2-OP for people aged 70 years or older. These models are calibrated in clusters of countries classified as low, moderate, high, and very high CVD risk, based on national mortality rates [33]. Risk scores are designed to provide more tailored preventive strategies that align with an individual’s risk profile, facilitating shared decision-making to ensure that preventive measures align with the patient’s preferences. Conversely, more precise risk stratification can lead to better-targeted interventions, potentially improving CV outcomes.

An individual’s estimated risk level must then be translated into treatment thresholds, based on LDL-C levels and according to the specific risk class (low-to-moderate, high, very high). The cut-off risk levels for these classes are numerically different for various age groups to avoid undertreatment in the young and overtreatment in older persons. Risk classes do not immediately translate into recommendations for starting drug treatment, and this decision should be individualized. In all age groups, consideration of risk modifiers, treatment benefit, comorbidities, frailty, and patient preferences may further guide treatment decisions; however, current guidelines still lack sufficiently detailed recommendations on how to incorporate these factors [11,15].

Patients with clinically established ASCVD are at very high risk of CV clinical events. In this class of very-high risk there are individuals with documented ASCVD, either clinical (e.g., previous acute coronary syndrome, chronic coronary syndromes, coronary revascularization or other arterial revascularization procedures; stroke; transient ischemic attack, peripheral arterial disease) or unequivocally on imaging (includes significant atherosclerotic plaques, >50% stenosis, on coronary angiography, computed tomography scan, carotid and femoral ultrasound, or markedly elevated coronary artery calcium (CAC) score). Other conditions classified as very high risk are diabetes mellitus with target organ damage or at least three major risk factors, early onset of type 1 diabetes of long duration (>20 years), severe chronic kidney disease (eGFR < 30 mL/min/1.73 m^2^), calculated SCORE2 or SCORE2-OP ≥20% and familial hypercholesterolemia with ASCVD or with another major risk factor [11,15]. Recently, a class of extreme risk was included for patients with ASCVD who experience recurrent vascular events while taking maximally tolerated statin-based therapy and for those with multiple territories arterial disease (e.g., coronary and peripheral) [15].

CVD risk may also be expressed from a lifetime perspective, for example, using the LIFE-CVD (LIFEtime-perspective CardioVascular Disease) calculator, identifying high-risk individuals both in the short and long term [34]. Such models consider competing risks from other diseases over the remaining expected lifespan, providing a more comprehensive and individualized assessment. By offering a lifetime perspective, tools like LIFE-CVD can support personalized preventive strategies, helping clinicians and patients to adopt informed decisions aiming the reduction in long-term CV risk. One of the limitations of the conventional risk scores is the fact that risk calculators usually indicate low percentages, which are not clearly understood by the patients, contributing to non-adherence to lifestyle and pharmacological interventions. Additionally, lifetime calculators lack guidance on cut-offs for more intensive treatment goals. Therefore, this concept tried to estimate CV risk not for the range of 10 years, over lifetime horizon [35].

## 4. Critical Gaps: Implementation Failures and Emerging Risks

### 4.1. Neglected and Emerging Risk Factors

Beyond the traditional well-established risk factors, there are additional, often neglected, CV risk markers that are also associated with the development of CVD. Obesity, unhealthy dietary patterns, physical inactivity and sleep disturbances increase the risk of CVD and should be carefully valued during risk estimation. However, despite robust evidence, these characteristics remain largely undervalued and continue to exhibit alarming prevalence among the general population.

Overweight and Obesity: A study analyzing the worldwide trends in underweight and obesity from 1990 to 2022 showed that the combined burden of these conditions has risen in most countries, driven by an increase in obesity, while underweight and thinness remain prevalent in south Asia and parts of Africa. As severe undernutrition persists in some regions, it is crucial to promote policies both to improve access to healthy diets and prevent obesity [36]. According to the ESC guidelines, a Mediterranean or similar diet is recommended to lower the risk of CVD, emphasizing the replacement of saturated fats with unsaturated fats and a reduction in salt intake to help control blood pressure. A more plant-based dietary pattern, rich in fiber and including whole grains, fruits, vegetables, pulses, and nuts, should be prioritized, while alcohol consumption should be limited, while fish, preferably fatty, should be eaten at least once a week and processed meat intake restricted. Additionally, consumption of free sugars, particularly sugar-sweetened beverages, should be kept below 10% of total energy intake [11,37].

Greater adherence to a Mediterranean diet has been consistently associated with a lower risk of CVD and reduced all-cause mortality, highlighting its role as a cornerstone of healthy dietary patterns for long-term health [38]. Barriers to adopting a heart-healthy diet must be assessed during CV risk assessment, including food access and economic factors. These factors may be particularly relevant to persons from vulnerable populations, such as individuals living in either inner-city or rural environments, those at socioeconomic disadvantage, and of advanced age. In the context of obesity management, important challenges persist, notably restricted access to treatment and the high cost of recently introduced pharmacotherapies. Nevertheless, novel anti-obesity agents (semaglutide and tirzepatide) have demonstrated significant CV benefits, achieving reductions of up to 20% in major adverse CV events among individuals with obesity and established CVD. The mechanisms appear to extend beyond weight loss, suggesting direct protective effects on cardiac tissue and metabolic processes [39,40].

Physical Inactivity: Physical inactivity is recognized as the fourth leading risk factor for global mortality, accounting for 6% of deaths worldwide and being a major contributor to the development of chronic and non-communicable diseases, including CVD, cancer, respiratory and mental health disorders. It is estimated that physical inactivity is responsible to 6% of the global burden of CAD, 7% of type 2 diabetes and 9% of premature mortality [41]. Beyond this clinical impact, physical inactivity is also associated with huge direct and indirect healthcare costs related to the management of these diseases [42,43]. Despite the well-known relationship between physical inactivity and CVD, its global prevalence among adults is nearly one-third (31%; about 1.8 billion people) with a constant increased rate since 2010. There are significant differences by gender, with women being less active than men, and by age, with increased rate by age after 60, but among adolescents the prevalence of physical inactivity is concerningly high (approximately 81%) [44].

The recent published RADICAL Study evaluated the prevalence of self-reported CV risk factors in individuals without known CVD using a digital tool, revealing a high prevalence of risk factors in a sample of 4149 individuals aged 40–69 years without known CVD. Beyond the relevance of traditional risk factors, such as hypercholesterolemia and hypertension, the results were concerning regarding physical activity (58.4%), obesity (19.4%), composite dietary risk factors that included combination of daily red meat consumption, excess salt and lack of fruits and vegetables (12.1%) and sleeping habits (33.7% sleep less than 7 h/night). Remarkably, 89.9% of participants reported at least one of the eight CV risk factors investigated [45].

Impaired sleep: An undervalued factor in clinical practice is short sleep duration and poor sleep quality, that have been associated with higher incidence of hypertension, obesity, type 2 diabetes, and ASCVD. This data supports the inclusion of sleep assessment as a routine component of CV risk evaluation. Overall, these findings illustrate the clustering of multiple lifestyle-related risk factors in apparently healthy individuals and highlight the implementation gap between guideline recommendations and real-world behavior.

Emerging non-conventional factors have been increasingly recognized as potentially contributing to CVD. However, many of these factors still lack robust evidence and remain largely neglected in routine risk assessment, underscoring the need for further research and integration into preventive strategies [46]. These emerging determinants frequently coexist with traditional risk factors, leading to a compounded and often multiplicative increase in CV risk, reinforcing the need for integrated population-level policies in parallel with individual-level interventions.

### 4.2. Established and Emerging Risk Markers

CV risk can be modified by several factors and clinical conditions, including genetic predispositions, inflammatory mediators and critically important lifestyle factors such as diet and physical activity [47]. In addition, circulating biomarkers, imaging findings, environmental and psychosocial characteristics can further refine individual risk estimates. As highlighted in the 2025 ESC guidelines, CV risk derived from traditional risk scores may be enhanced through the integration of these specific risk modifiers, allowing for more precise and personalized prevention strategies. By integrating these factors, it is possible to identify the individuals whose actual risk is higher or lower than estimated by conventional risk scores, improving risk discrimination [11,15]. This is particularly important for individuals around treatment decision thresholds, where risk modifiers can change management.

A useful risk modifier should improve risk prediction, enhance both discrimination and reclassification, demonstrate a clear public health impact, be practical for routine clinical use and provide information not only on how risk increases with adverse findings but also on how it decreases when results are favorable. Additionally, the evidence supporting the markers should be robust and not substantially influenced by publication bias [11,15]. Among the multiple markers proposed, the robustness of evidence varies, although some are already included in guidelines. Table 1 presents the main neglected risk factors and additional conditions that should be considered as CV risk markers.

Imaging: One of the most relevant risk markers is the CAC score [11,48,49]. CAC score consistently enhances CV risk discrimination and reclassification, particularly useful in cases around treatment decision thresholds. Higher CAC values are strongly associated with an increased risk of CV events in individuals without clinical ASCVD [48,50]. This marker predicts risk with the same magnitude of effect in all races, age groups, and both sexes, being one of the most relevant markers for predicting the risk of ASCVD [51]. Individuals with very high CAC scores have a markedly elevated risk for CV events, including mortality, compared with those who have lower CAC levels, with a similar to or may even exceed rate that of patients with established ASCVD [52]. Otherwise, a CAC score of zero identifies individuals at low to moderate estimated risk. In individuals classified at high risk by clinical scores, a zero CAC score confers better survival than those at low to intermediate risk but with any CAC [53]. In this context, incorporating CAC score improves the accuracy of CVD risk prediction when added to SCORE2 [54]. Nevertheless, considerations such as cost, radiation exposure, and limited availability must be considered before widespread adoption across all risk groups. More recent guidelines explicitly recommend using subclinical coronary atherosclerosis or high CAC score as an actionable risk modifier, especially in those with moderate risk or close to intervention thresholds.

When CAC score is unavailable or not feasible, the presence of arterial plaque in other vascular territories, such as carotid or femoral arteries, detected by imaging, should also be considered as a risk modifier, reflecting the risk associated with subclinical atherosclerosis beyond coronary arteries [55]. Other evaluations that can improve CV risk classification are the measurement of arterial stiffness using either aortic pulse wave velocity or arterial augmentation index, and the ankle brachial index [11]. These vascular markers reflect aortic and peripheral atherosclerosis, providing pathophysiological information that is complementary to traditional risk factors.

Biomarkers: Other objective markers have gained progressive importance in the last years, potentially playing a pivotal role as adjunct for global risk assessment in primary CV prevention. Among the more studied are lipoprotein (Lp(a)) and high-sensitivity C-reactive protein (hs-CRP). Lp(a) is a proinflammatory and potent atherogenic lipoprotein, genetically determined, independent, and causal risk factor for ASCVD. A value of Lp(a) > 50 mg/dL (>105 nmol/L) should be considered as a CV risk-enhancing factor in adults, given the robust evidence for its incremental predictive value. Persistent higher hs-CRP (>2 mg/L) is a widely recognized marker of systemic inflammation, often used in clinical settings to assess inflammation-related ASCVD risk, predicting CV events and all-cause mortality at long-term follow-up [56,57]. Furthermore, these markers can independently and synergistically contribute to an increased risk for CV events [58]. Despite their prognostic value, current evidence supports their use mainly for risk refinement rather than for population-wide screening. Specific Lp(a)-lowering therapies are still under development. Among additional biomarkers that have been linked to increased CVD risk are uric acid, homocysteine, and vitamin D. However, as the available evidence is largely observational, with inconsistent results and absence of demonstration of clinical benefit from routine measurement, current guidelines do not recommend their use for standard CV risk assessment [14].

Mental health: Mental health disorders and CVD are closely linked, with a bidirectional relationship. Psychosocial stress, including stress symptoms as well as stressors such as work stress (including burnout and unemployment), family issues, impaired social relationships, socioeconomic stress, financial pressures, loneliness/social deprivation and critical life events, increase the risk for the development and progression of ASCVD. Several mental health conditions such as depression, anxiety, post-traumatic stress disorders and personality traits like Type D personality, are also linked to poorer CV prognosis. Additionally, these conditions negatively affect adherence, prognosis, and quality of life in patients with established CVD [59,60]. In this setting, routine assessment for mental disorders is recommended in CV care using validated tools, while management should involve a multidisciplinary approach to provide integrated and patient-centered care. Promoting mental well-being and addressing psychosocial stressors are essential components of CV prevention, being effective communication crucial to address emotional factors influencing risk perception [61].

Demographic and socioeconomic data: In terms of ethnicity, individuals from southern Asia, especially from countries such as India, Pakistan and Bangladesh, have higher rates of several CV risk factors, being recommended the application of correction factors, when assessing CVD risk with risk calculators. Multiplication of calculated risk by the relative risk for specific ethnic subgroups should be considered [11]. Socioeconomic determinants are major markers of increased risk, as evident in the higher rate of CV events in low-income populations. Even in high-income countries, socioeconomic disparities strongly influence CV risk [62]. Socioeconomic inequalities and educational status are strong determinants of CVD risk, while health literacy should be regularly assessed to maximize the effectiveness of the recommendations. Incorporating social risk into prevention strategies is essential to avoid widening existing health disparities [11]. The inclusion of the postcode in CV risk calculation tables aims to classify socioeconomic status according to place of residence, as occurs with PREVENT (Predicting Risk of Cardiovascular Disease Events) risk equation, already recommended by the new American College of Cardiology/American Heart Association guidelines for the prevention, detection, evaluation, and management of high blood pressure in adults [63,64].

Environmental factors: Environmental exposures, particularly air pollution, are increasingly recognized as important CVD risk markers. Air pollution with relevance for fine particulate matter is linked to approximately 8.3 million deaths annually, over half from CVD, and even levels below regulatory limits promote atherosclerosis, vascular dysfunction, and cardiac events [11]. Emerging threats such as micro- and nano-plastics, along with noise pollution, heat extremes, toxic chemicals, and light pollution, also contribute to vascular injury through oxidative stress, inflammation, and circadian disruption. Climate change further amplifies these risks, especially in vulnerable settings [65]. Despite growing evidence, these exposures are not yet routinely captured in individual risk calculators, representing an important blind spot in current assessment tools.

Genetics and family background: Family history of premature CVD is associated with higher risk, reflecting the genetic and environmental interplay [66]. The age threshold for defining premature CVD is before age 55 years in male first-degree relatives and before 65 years in female first-degree relatives., comprising parents and full siblings. Documented clinical CAD, revascularization, stroke, sudden death, familial hypercholesterolemia or other genetic lipid disorders strengthens this risk. Genetics, especially polygenic risk scores, are being explored as potentially valuable tools for enhancing CVD risk prediction. Integrating polygenic risk scores with conventional risk factors can improve risk stratification, identify individuals at higher risk, and support personalized prevention strategies. However, the evidence shows only modest incremental accuracy and insufficient consensus for their widespread use in routine clinical practice [67].

Gut microbiome: The interplay between the gut microbiome and several diseases, including ASCVD, has been increasingly recognized. An imbalance in gut microbial communities (dysbiosis) can promote systemic inflammation through multiple pathways and contribute to atherosclerosis development. Gut microbiota modulation, especially via dietary interventions, offers a promising strategy to reduce CV risk by targeting metabolic and inflammatory mechanisms, warranting more in-depth investigation in future research [68,69]. However, current data are still insufficient to support routine microbiome-based testing in CV risk stratification.

Inflammatory diseases: Several chronic inflammatory-related diseases increase CV risk through systemic inflammation, immune dysregulation, and promotion of atherosclerosis and thrombosis. The association between CVD and these conditions is bidirectional, including rheumatoid arthritis, systemic lupus erythematosus, psoriasis, inflammatory bowel diseases (Crohn’s disease and ulcerative colitis) and human immunodeficiency virus infection [70].

Sleep disturbances: Sleep disorders, including obstructive sleep apnea (OSA), insomnia, restless leg syndrome, narcolepsy and circadian rhythm disturbances, are significantly associated with CVD. These disorders promote systemic inflammation, sympathetic nervous system activation, oxidative stress, and metabolic dysregulation, leading to conditions such as hypertension, atherosclerosis, arrhythmias, and heart failure. Effective management, including treatments like continuous positive airway pressure for OSA and cognitive-behavioral therapy for insomnia, can help to mitigate these risks, but adherence and awareness remain challenges [71].

Other potential risk markers: Frailty is another potential risk modifier that has been associated to an increased risk of mortality and several CVDs. Furthermore, hypertensive disorders of pregnancy (2–4-fold higher long-term risk of hypertension, CAD, heart failure, and stroke) and premature menopause (metabolic, endothelial, and lipid alterations) are also referred as conditions to be valued in this setting [11,15,72]. These sex-specific and age-related factors are particularly important for tailoring preventive strategies in women and older adults and should not be overlooked in routine risk assessment.

Beyond risk modifiers, several clinical conditions (e.g., chronic kidney disease, heart failure, cancer and chronic lung disease) are strongly associated with increased CVD risk and poorer prognosis. Effective managing of these conditions can provide synergistic reductions in overall CV risk and disease burden, highlighting the value of a holistic and multidisciplinary prevention approach as emphasized in the latest guidelines [11].

### 4.3. Beyond the Guidelines: Failures in Implantation and Adherence

**Figure 2:**
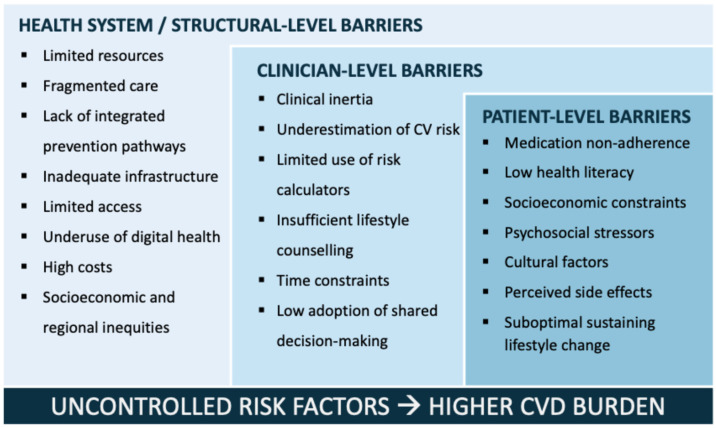
Multilevel (health-system/structural-, clinician-, and patient-level) barriers to effective CV prevention implementation.

Despite the availability of robust and evidence-based guidelines, a substantial evidence-practice gap persists in CV prevention. Many high-risk individuals continue to fall short of recommended targets for blood pressure, LDL-C, glycemic control, weight, and lifestyle change. These shortcomings arise from complex, multilevel barriers that limit the translation of guidelines into real-world clinical practice [20,73]. Figure 2 presents the multilevel barriers (health-system/structural-, clinician-, and patient-level) to the implementation of effective CV prevention. These interconnected obstacles ultimately contribute to persistent uncontrolled risk factors and a sustained global burden of CVD.

At health system level, several obstacles contribute to poor implementation of guidelines. These include limited resources, inadequate or fragmented healthcare infrastructure, insufficient integration between primary care and specialty services, and unequal access to both pharmacologic and non-pharmacologic interventions [74]. In many healthcare settings, preventive care lacks prioritization, with inconsistent reimbursement policies and limited availability of structured prevention programs. The underuse of digital health technologies and risk assessment interfaces further reduces opportunities for continuous monitoring and follow-up. Vulnerable populations and individuals with multimorbidity are disproportionately affected by these structural deficiencies. In fact, regional and socioeconomic disparities further exacerbate implementation gaps. Populations in low- and middle-income countries, and underserved communities within high-income settings, face disproportionate barriers related to availability of care and poor healthcare infrastructure [75,76]. Addressing these inequities requires culturally adapted strategies, enhanced policy support, and coordinated care models across sectors. Development of telehealth and community-based services can play a critical role in expanding access and ensuring continuity of preventive care.

Guideline implementation is strongly influenced by clinician-related factors. Among these factors, clinical inertia remains a major barrier, corresponding to the failure to initiate or intensify therapy despite unmet targets, often driven by time constraints, competing clinical demands, and therapeutic complexity [62,74]. Underestimation of CV risk, insufficient use of risk calculators, low adoption of shared decision-making, and inadequate training or familiarity with updated recommendations, all contribute to inconsistent practice. In addition, limited consultation time often results in superficial lifestyle counselling that is insufficient to support sustained behavioral change.

Patient adherence represents one of the most persistent challenges in CV prevention. Non-adherence to medications is common, frequently worsened by polypharmacy, perceived or actual side effects, high out-of-pocket costs, and limited understanding of treatment benefits. Lifestyle recommendations are also difficult to maintain in the long term due to psychosocial stressors, cultural factors, socioeconomic constraints, low health literacy, and competing life priorities [77]. These barriers directly contribute to suboptimal risk factor control and higher rates of adverse CV events.

Effective risk communication is essential for informed decision-making, yet patient-clinician interactions are often compromised by cognitive biases, emotional responses, and misinterpretation of numerical risk estimates. Patient–doctor interactions are complex, and effective risk communication remains a challenge, without a standard strategy or optimal approach that should be individualized according to the patient’s preferences, level of understanding and health literacy [78]. A recent meta-analysis showed that communicating CVD risk information, regardless of the method, reduced the overall risk factors and enhanced patients’ self-perceived risk [79]. Ensuring that patients truly understand their CV risk, the anticipated benefits of interventions, and the trade-offs associated with treatment is critical. Approaches such as risk age (or heart age) have shown promise in enhancing comprehension, particularly among younger individuals whose absolute risk may appear low despite high relative lifetime risk. This approach has proven particularly useful in enhancing risk communication within disease risk calculators, but there is also a lack of standardized materials for patient communication endorsed by national societies [80].

Ultimately, the central problem in CV prevention is not the absence of scientific knowledge, but the failure to translate evidence into sustainable, equitable, and effective practice [74]. Addressing implementation failures requires a shift from guideline prescription toward personalized engagement strategies, informed by behavioral economics, patient empowerment, and culturally appropriate communication. At the system level, integrating digital innovations, simplifying care pathways, and embedding principles of implementation science can help create environments where adherence is supported rather than merely expected [62]. Only coordinated and aligned efforts across patient, clinician, and health-system domains can enable CV prevention to achieve its full potential in reducing the global burden of CVD.

## 5. Novel Strategies and Future Directions

Despite substantial advances in CV prevention, significant gaps remain in guideline implementation, risk communication, and equitable access to care. Addressing these challenges requires a multifaceted approach that integrates patient-centered strategies, shared decision-making, and interventions tailored to individual characteristics such as sex, age, race, mental health, and socioeconomic context. Improving risk communication, fostering shared decision-making, optimizing screening strategies, and defining effective mental health interventions within CV care are critical priorities for future research and practice [62,74].

**Figure 3:**
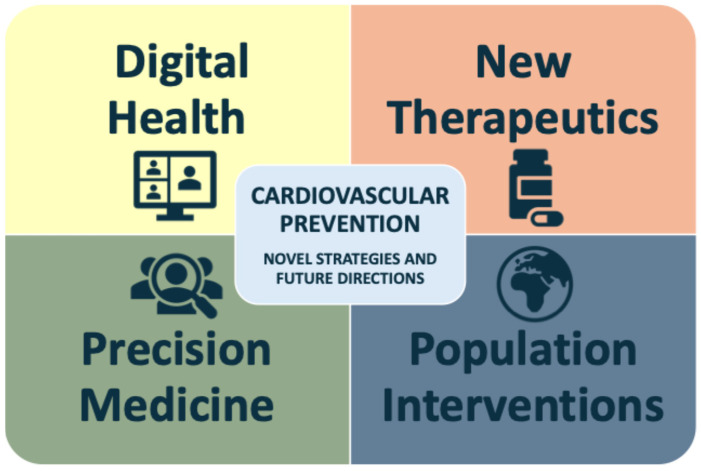
Main emerging strategies to enhance future CV prevention (digital health, precision medicine, new therapeutics and population interventions).

Innovative approaches, including genetic testing, advanced risk stratification methodologies, digital health technologies, emerging pharmacological and behavioral interventions, are being explored to enhance early detection, personalize therapy, and improve long-term adherence. A static model is unlikely to meet the several needs of individuals at increased CV risk; therefore, tailoring interventions to individual circumstances is essential for maximizing engagement and achieving meaningful reductions in disease burden [81,82]. Figure 3 illustrates the main emerging strategies to enhance future CV prevention. Strengthening CV health literacy and establishing a structured shared decision-making process are fundamental to the success of these approaches.

### 5.1. Digital Health and Technology

The digital revolution is reshaping CV prevention, offering unprecedented opportunities for early detection, risk stratification, and patient-centered care. Wearable devices, mobile health applications, and telemedicine platforms allow continuous remote monitoring of vital signs, physical activity, sleep patterns, and other behaviors, providing real-time data that can facilitate timely and personalized interventions [12,14]. For example, wearable devices can continuously monitor heart rate, blood pressure or physical activity and automatically alert clinicians if abnormal patterns are detected, prompting early evaluation and intervention. Otherwise, digital tools enable earlier identification of subclinical disease, including asymptomatic atherosclerosis and vascular dysfunction, which is critical for initiating preventive measures [83,84].

AI-based and machine learning tools show promise to further enhance CV risk prediction by integrating multimodal data, including imaging, biomarkers and lifestyle factors, to generate individualized risk profiles. Automated algorithms can help identify individuals at particularly high risk, suggest optimized treatment pathways and track adherence to pharmacological and lifestyle interventions. Mobile health applications can deliver personalized lifestyle recommendations, such as tailored exercise programs, dietary guidance and reminders for medication adherence, which can be integrated into clinical decision-making platforms to support timely preventive actions. More recently, large language models and other generative AI systems have emerged as potential tools to synthesize complex information, support patient education and assist clinicians with documentation and guideline navigation, although their clinical utility in CV prevention remains to be robustly established [85]. Digital health technologies additionally reduce administrative tasks, enable clinicians to allocate more time to direct patient care [86].

Importantly, these tools also have the potential to reduce, but also to exacerbate, inequities in healthcare access. Telemedicine and mobile platforms can extend preventive services to underserved populations, including rural communities and low- and middle-income countries, while educational programs in digital applications can improve health literacy and facilitate the adoption of lifestyle modifications. By providing tailored feedback and interactive coaching, digital solutions may enhance patient engagement and foster sustained behavioral change, addressing a critical gap in preventive care [87].

Despite these advances, challenges persist, including infrastructure requirements, access disparities, digital literacy, data privacy, cybersecurity, and integration into clinical workflows. Evidence for long-term effectiveness and cost-efficiency is still evolving. Looking forward, innovative applications such as virtual patient twins, predictive analytics, and AI-driven modeling have the potential to further transform CV prevention by simulating individualized disease trajectories and enabling the optimization of preventive strategies at both patient and population levels [88,89]. However, these developments will require rigorous validation, robust governance frameworks and careful evaluation of their impact on equity and quality of care.

### 5.2. New Therapeutics and Targets

Recent advances in pharmacotherapy have significantly expanded the toolbox for CV prevention, including drug classes, such as PCSK9 inhibitors and RNA-based therapies, offering potent reductions in LDL-C and other atherogenic lipoproteins. Other emerging therapeutic targets, such as Lp(a), chronic inflammation (e.g., IL-1 inhibitors), and gut microbiome modulation, are under investigation, potentially opening new avenues to reduce residual CV risk beyond traditional lipid-lowering and blood pressure management [90,91]. For example, once approved, Lp(a)-lowering therapies could be incorporated into clinical pathways by prioritizing individuals identified as high-risk through polygenic risk scores, family history, or elevated baseline Lp(a) levels, enabling targeted preventive interventions. Similarly, anti-inflammatory therapies could be guided by biomarker profiling (e.g., hs-CRP) to identify patients who would benefit most from additional therapy.

Combination therapies targeting multiple pathways, patient-tailored regimens, precision dosing, pharmacogenomic-guided therapy, and repurposing of existing medications are increasingly emphasized, reflecting a shift toward holistic, mechanism-driven prevention. These strategies also hold promise for optimizing individual responses and minimizing adverse effects. The polypill strategy could help reduce the growing global burden of CVD by providing a simple, evidence-based, and innovative preventive approach [55]. This approach, which combines multiple preventive medications into a single pill, can be implemented in primary care or community health programs to simplify multi-drug regimens, particularly in low-resource settings, enhancing adherence and reducing the overall burden of CVD. Similarly, pharmacogenomic-guided therapy allows clinicians to tailor drug selection and dosing to a patient’s genetic profile, potentially improving efficacy and reducing the rate of adverse effects.

Despite these developments, challenges persist regarding cost, long-term safety, accessibility, and equitable distribution of these therapies.

### 5.3. The Era of Precision Medicine

Precision medicine represents a transformative approach in CV prevention, leveraging a combination of genetic, molecular, imaging, and lifestyle data to tailor risk assessment and interventions. Polygenic risk scores could enable early identification of high-risk individuals, even among younger populations with low absolute risk, facilitating timely preventive measures. Biomarkers and advanced imaging, including CAC score and functional vascular assessments, provide comprehensive insights into subclinical atherosclerosis and help guide individualized therapy. CAC score can guide decisions on lipid-lowering therapy initiation or intensity for patients at intermediate risk. Furthermore, integrating additional risk-modifying characteristics that are not included in existing CVD risk models can improve the accuracy and applicability of risk prediction, enhancing personalized preventive strategies [92]. Multimodal dashboards that combine laboratory results, imaging data and lifestyle information can help clinicians and patients make shared decisions and implement personalized preventive strategies.

Integrating these tools enables clinicians to deliver highly targeted interventions, optimizing benefit-risk ratios for each patient. Precision medicine also enhances decision-making by incorporating patient preferences, comorbidities, and socio-demographic factors, fostering adherence and engagement. However, widespread adoption faces barriers including cost, ethical considerations, accessibility, and the need for clinician training to interpret and apply complex datasets [93]. Ensuring equitable application is essential to avoid exacerbating existing health disparities.

### 5.4. Policy and Population Health Interventions

Population-level strategies and health policies remain cornerstones of CV prevention. Robust tobacco control policies, including taxation, advertising restrictions, and limits on industry influence, have significantly reduced smoking prevalence and CV morbidity. Additional population-based strategies, such as promoting sugar-sweetened beverage taxes and mandatory front-of-package food labeling have been shown to reduce consumption of unhealthy foods and improve population-level lipid profiles. Other interventions, such as the development of salt reduction programs, and school-based nutrition and physical activity initiatives, further contribute to healthier lifestyles and lower CV risk. Policies should prioritize equity by addressing social, economic, and cultural determinants of health, thereby reducing disparities in CV risk and outcomes [62,94].

Digital health technologies, telemedicine, and community-based interventions offer opportunities to expand access to preventive care in underserved populations. Examples of community-based initiatives include free physical activity programs, mobile health screening units, and public education campaigns, all of which can enhance adherence to preventive behaviors, particularly in rural or resource-limited settings. Governance, regulatory frameworks, and reimbursement strategies are essential to support sustainable implementation of these innovations. Global collaboration between international agencies, governments, and healthcare systems is critical to align strategies, monitor impact, and scale effective interventions [81,94]. Looking ahead, policies that integrate precision public health and adaptive risk-reduction programs have the potential to optimize CV prevention at both individual and population levels while addressing systemic inequities and resource limitations.

## 6. Conclusions

Despite significant developments in CV prevention, persistent gaps remain in the real-world effective implementation of clinical guidelines. The integration of traditional and emerging risk factors with innovative methodologies, including digital health, precision medicine, novel pharmacotherapies, and population-level interventions, offers promising avenues for earlier detection, tailored treatments and enhanced adherence. Overcoming system, clinician and patient-level barriers, while simultaneously fostering shared decision-making and equitable healthcare access, is crucial to maximizing preventive outcomes. By anchoring these approaches within implementation science and health equity frameworks, CV prevention can progress towards a more precise, inclusive, and globally impactful paradigm, with the potential to reduce the global burden of CVD.

## Figures and Tables

**Table 1:** Main neglected risk factors and additional conditions that should be considered as CV risk markers.

Domain	Risk Factor/Marker	Anormal Finding or High-Risk Profiles
Demographic	Family history of premature CVD	First degree-relatives: men: <55 years; women: <65 years (clinical CAD, arterial revascularization, stroke, sudden CV death, familial hypercholesterolemia or other genetic lipid disorders).
	Ethnicity	South Asian ancestry (e.g., India, Pakistan, Bangladesh)
Psychosocial	Stressors	Work stress, burnout, unemployment, lifetime discrimination, family issues, impaired social relationships, social deprivation, low socioeconomic status, loneliness/social isolation.
	Mental health conditions	Anxiety, depression, post-traumatic stress disorders, personality traits (e.g., Type D personality), substances abuse (e.g., alcohol, illicit drugs and other toxics substances).
Lifestyle and Behavioral	Overweight and Obesity	BMI: overweight BMI 25.0–29.9 kg/m^2^; obesity ≥30 kg/m^2^. Waist circumference >102 cm in men, >88 cm in women (associated with visceral adiposity).
	Physical inactivity	<150 min of moderate-intensity PA or <75 min of vigorous-intensity PA per week.
	Sleep disorders	OSA, insomnia, restless leg syndrome, narcolepsy, and circadian rhythm disturbances.
Immune-mediated	Inflammatory	Rheumatoid arthritis, systemic lupus erythematosus, psoriasis, inflammatory bowel diseases (Crohn’s disease and ulcerative colitis).
	Infectious	HIV infection
Sex-specific	Premature menopause	Permanent cessation of ovarian function before 40 years old.
	HT disorders of pregnancy	Gestational hypertension, preeclampsia, eclampsia.
Environmental exposures	Chemical agents and pollutants	Air pollution, micro- and nano-plastics, heavy metals, toxic chemicals.
	Physical and climate-related	Noise pollution, heat extremes, light pollution, climate change.
Imaging and Biomarkers	CAC	101–300 moderate calcification >300 severe calcification (high risk of ASCVD)
	hs-CRP	>2 mg/L (values >10 mg/L should prompt investigation for other inflammatory or infectious conditions as they may not reflect CV risk alone).
	Lp(a)	>50 mg/d (>105 nmol/L); >180 mg/dL (>430 nmol/L) lifetime risk comparable to untreated heterozygous FH).

ASCVD: Atherosclerotic Cardiovascular Disease; CAC: Coronary Artery Calcium; CAD: Coronary Artery Disease; CV: Cardiovascular; CVD: Cardiovascular Disease; FH: Familial Hypercholesterolemia; HT: Hypertensive; HIV: Human Immunodeficiency Virus; hs-CRP: high-sensitivity C-reactive protein; Lp(a): Lipoprotein(a); OSA: Obstructive Sleep Apnea; PA: Physical Activity.

## Data Availability

The original contributions presented in this study are included in the article. Further inquiries can be directed to the corresponding author.
